# Supplementary material for: Ni-Catalyzed Enantioselective Intramolecular Mizoroki–Heck Reaction for the Synthesis of Phenanthridinone Derivatives
Source: J Org Chem. 2023 Jun 15;88(13):8203–26. doi: 10.1021/acs.joc.3c00202 (PMC10337041; doi:10.1021/acs.joc.3c00202)

YK-114-092 F7 CDCl3 H
PROTON CDCl3 {D:\nmrusers\malachowski} BM 12

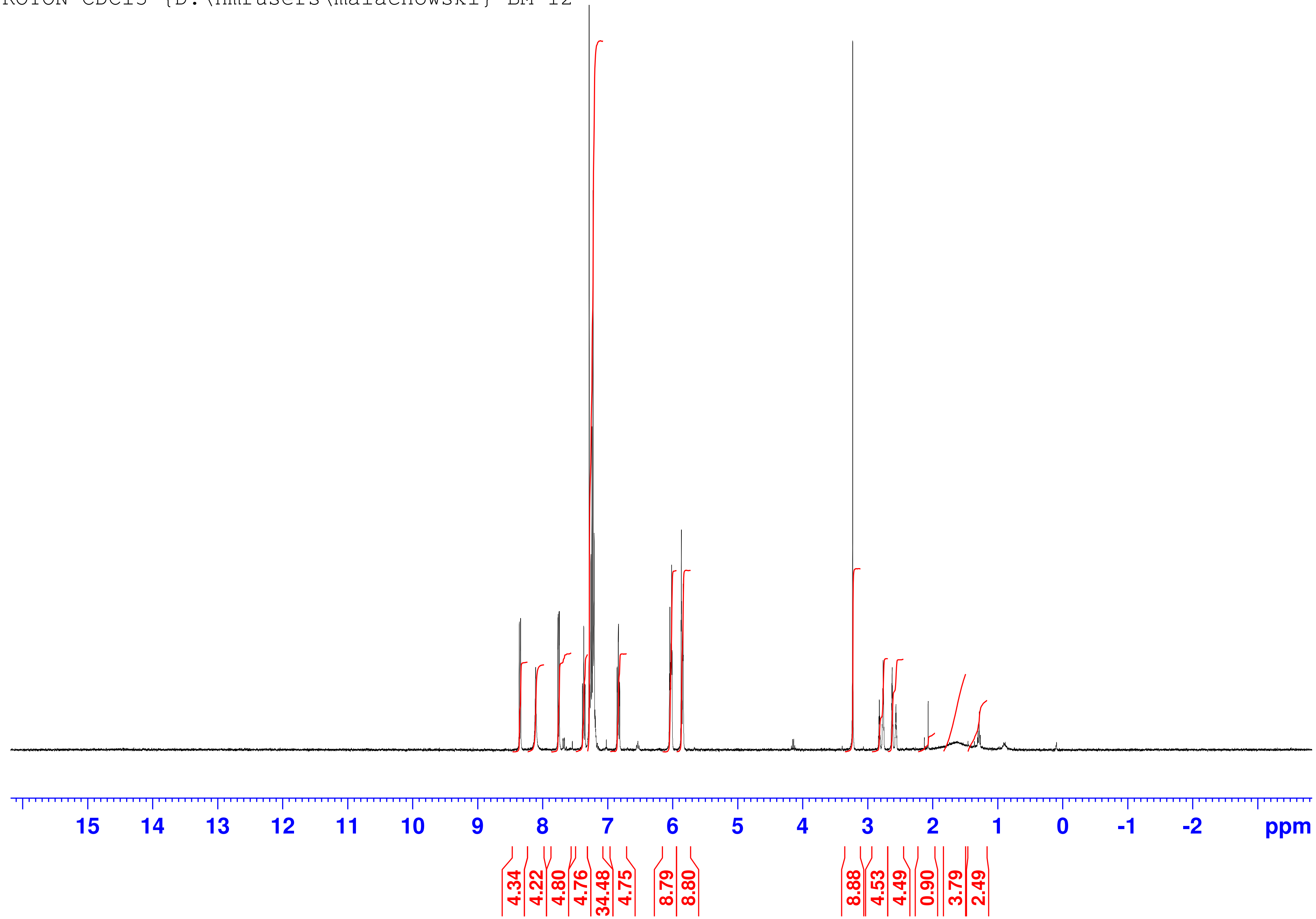

Supplement: Supplementary file 2 — jo3c00202_si_002.zip [file jo3c00202_si_002.zip › Amides(N-H and N-Me)/S2h-I Bn-NH-ArI/1H Bn-NH-ArI S2h-I/1/pdata/1/email_YK-114-092 F7 CDCl3 H_1_1.pdf]
